# A case of rabies in a Kano brown doe

**DOI:** 10.1002/ccr3.1821

**Published:** 2018-09-23

**Authors:** Bilkisu Yunusa Kaltungo, Solomon Wuyah Audu, Ibrahim Salisu, Solomon Olu Okaiyeto, Balarabe Magaji Jahun

**Affiliations:** ^1^ Veterinary Teaching Hospital Ahmadu Bello University Zaria Nigeria; ^2^ Department of Veterinary Medicine Ahmadu Bello University Zaria Nigeria

**Keywords:** goat, Kano brown, rabies

## Abstract

This manuscript aims at creating awareness especially to livestock farmers and veterinarians/clinicians that rabies occurs in livestock species. Furthermore, clinicians should note that the viral load in dog bite cases by rabid dog can be reduced by vigorous washing and disinfection which may ultimately delay the clinical manifestation of rabies.

## 1 INTRODUCTION

The upsurge in the reports of rabies in Nigerian livestock population is a potential threat to livestock handlers. A Kano brown doe was investigated for rabies after being bitten by a known rabid dog. The goat died after 37 days of quarantine and tested positive for rabies using rapid immunochromatographic test and direct fluorescent antibody test. Routine vaccination of dogs against rabies should be enforced in endemic countries.

Rabies, otherwise known as “ara nkita” in Igbo, “digbolugi” in Yoruba, “haukan kare” in Hausa, “ginnaji” in Fulfude, and “Idat ebua” in Efik by some Nigerian languages, is a viral disease of all warm‐blooded animals. The disease has generally been underreported especially in endemic countries. Despite this, it has been reported to cause about 59 000 humans deaths annually across Africa and Asia.^1^ In addition, economic costs due to rabies were estimated to be 8.6 billion USD annually.^1^ Naturally, its primary host is the domestic and wild dogs. However, other animals such as cats, foxes, and bats are also susceptible and have been incriminated as reservoir hosts which serve as sources of infection to humans and other animals including livestock.^2^ The incubation period of the disease is variable ranging from 5 days to 1 year depending on species involved, site of bite, and quantity of virus in the inoculum.^3^

Exposure to rabies in developing countries of Africa and Asia is mainly through bites from rabid dogs, and this has been reported to account for about 98% of confirmed human rabies cases.^1^, ^4^ Reports from China show that most livestock cases occur as spillovers from rabid carnivores.^5^ Initial clinical diagnosis of rabies in goats is often challenging because of similar clinical presentations with several diseases. Most often than not, presenting signs are excessive bleating or bellowing, anorexia, ataxia, tenesmus, neurological abnormalities, and lameness. However, excessive bleating is the most common sign in goats.^6^

Since the first report of human rabies in Nigeria in 1912 and in dogs in 1925, several researchers have reported rabies in man and in different species of animals.^2^, ^7^, ^8^, ^9^ These reports prove beyond doubt that rabies is endemic in Nigeria. Furthermore, in Nigeria, estimated the total death in domestic animals due to rabies to be 1000 per annum in the 1970s.^10^ This figure may have increased as evidenced by several recent reports on rabies in this group of animals.^6^

Reports on rabies in goats are rare. However, like all mammals, they are susceptible with the furious form most frequently manifested than the dumb form.^11^ The lack of awareness of rabies especially in domestic animals makes it a potential public health threat.^3^

This paper reports a case of rabies in a 3‐year‐old Kano Brown doe.

## 2 CASE PRESENTATION AND MANAGEMENT

**Figure 1:**
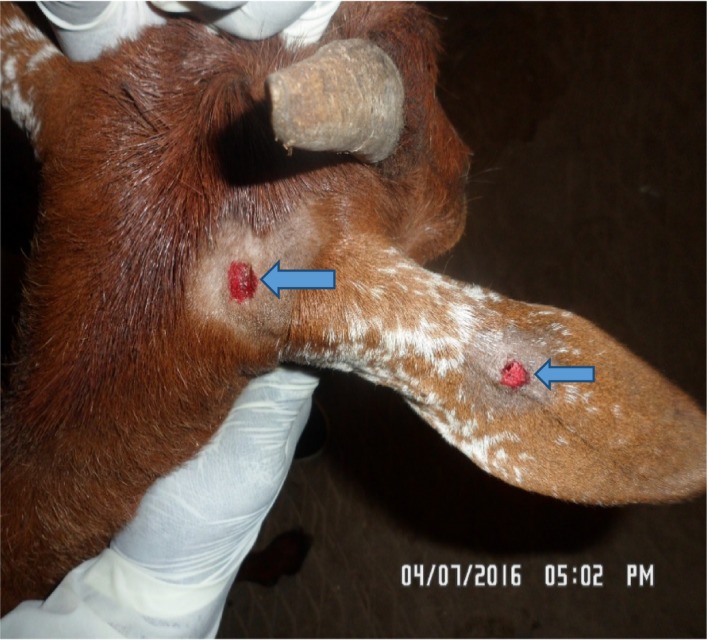
Dog bite wounds on the right pinna of a Kano‐Brown doe

A 3‐year‐old Kano Brown doe weighing 20 kg from a herd of six was presented to the Large Animal Clinic of the Veterinary Teaching Hospital (VTH), Ahmadu Bello University, Zaria, with the chief complaint of bite by a “mad dog” a few hours prior to presentation. History revealed that the dog bit and killed a goat kid in the same herd and two members of the household. The suspected dog belonged to a neighbor and had no history of vaccination against rabies. The dog was caught and presented to the Small Animal Unit of the same hospital where it was quarantined. It died after a couple of days, and its brain tested positive for rabies. The herd was managed on a semi‐intensive system. Upon physical examination, all vital parameters were within the normal range (temperature = 38.5°C, respiratory rate = 24 cycles per minute, and pulse rate = 75 beats per minute) and puncture bite wounds were observed at the base of the right ear and pinna (Figure 1). The area around the bite wounds was shaved and vigorously washed using a moderately hard brush with plenty of water under pressure and an antiseptic soap containing Chlorhexidine (Septol^®^, saverscart, Lagos State, Nigeria). Tetanus toxoid (1500 IU) and a long‐acting oxytetracycline (Samoxine^®^, Sam parmaceuticals, Kwara State, Nigeria) (@ 20 mg/kg/body weight) total dose 400 mg were then administered intramuscularly. Since there is currently no rabies vaccine available for livestock in Nigeria, the goat was quarantined in the goat pen within the hospital for close observation.

Thirty‐seven days into the period of quarantine, the goat was observed to exhibit some abnormal behaviors such as nibbling on the metal fence of the pen, foamy salivation, excessive bleating, and also could not eat or drink despite being offered palatable feed such as maize bran and groundnut leaves. It often attempts nibbling on the feed but cannot chew or swallow.

During the postmortem examination, the goat was decapitated and brain harvested and placed in two polythene bags using the method described by Kaplan and Koprowski (1980). The extracted brain samples were sent in ice‐packed containers to Virology Laboratory, Department of Veterinary Medicine and Viral Zoonoses Laboratory, Department of Veterinary Public Health and Preventive Medicine, Faculty of Veterinary Medicine, Ahmadu Bello University, Zaria, for rapid immunochromatographic test and direct fluorescent antibody test, respectively.

### 2.1 Rapid immunochromatographic diagnostic test

The lateral flow assay (LFA) using the rapid immunochromatographic diagnostic test (RIDT) kit for rabies antigen (BioNote, Inc. 2‐9, Seogu‐dong, Hwaseong‐si, Gyeonggi‐do, Korea). The test was conducted according to the manufacturer's recommendation.

This test is based on antigen‐antibody neutralization, and it involves the use of a gold‐labeled monoclonal antibody (MAb). Purified goat anti‐mouse IgG is immobilized in the control zone to capture unbound MAb, and purified anti‐nucleoproteins MAb are immobilized in the test zone of the nitrocellulose membrane. Once a sample is dropped into the sample well, it flows through the gold MAb pad, the test zone, and the control zone.

A 10% (w/v) homogenate of the brain sample (hippocampus, cerebellum, and brain stem) was prepared and collected by a sterile swab and then dipped in the supplied buffer assay and stirred to mix and ensure extraction. A sterile dropper is used to drip 2‐3 drops of the suspension into the sample well of the test cassette. Results were interpreted within 5‐10 minutes.

**Figure 2:**
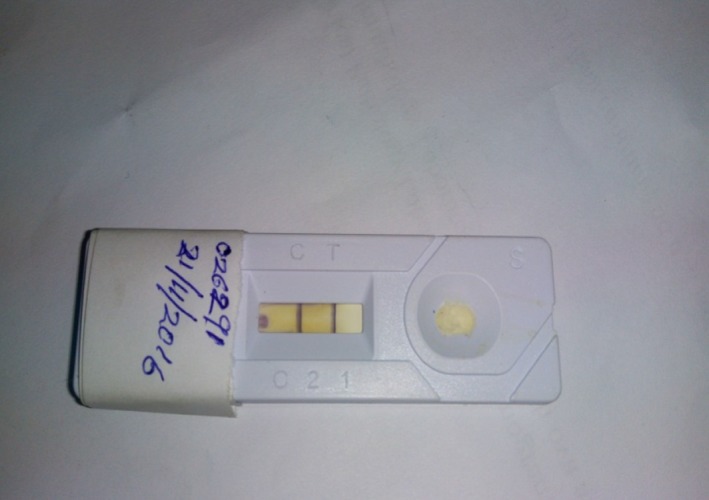
Rapid immunochromatographic test for rabies antigen showing positive result

The test result is considered positive on appearance of two purple colour bands (''C'') and test (''T'') within the test window (Figure 2). While it is negative when only a single purple band (''C'') appears within the test window. The test is considered invalid when the band at the control does not appear.

### 2.2 Direct fluorescent antibody test (DFAT)

A working dilution of 1:40 of the rabies direct titration fluorescent antibody assay was achieved following titration assay reagent (monoclonal antibody conjugate) by Fujirebio Inc. Diagnostic in accordance with manufacturer's recommendations and as described by Flamand et al.^12^ Briefly, a small fraction of the brain sample was smeared using wire loop on one part of a slide pressed on a disposable tissue to allow for an even spread and make the smear light and then air‐dried and fixed in cold acetone for one hour at −20^°^C. The slides were then air‐dried, and then, the rabies conjugate was applied to cover the smear and incubated for 30 minutes at 37°C in a humid chamber, after which excess conjugate was removed from the slides by rinsing it with 7.4 pH PBS solution about 3‐5 minutes and then allowed to air dry. Then, coverslips were mounted with buffered glycerol mounting medium, and the slides were examined using a Fluorescent microscope (Carl Zeiss, Germany) within 2 hours after staining.

**Figure 3:**
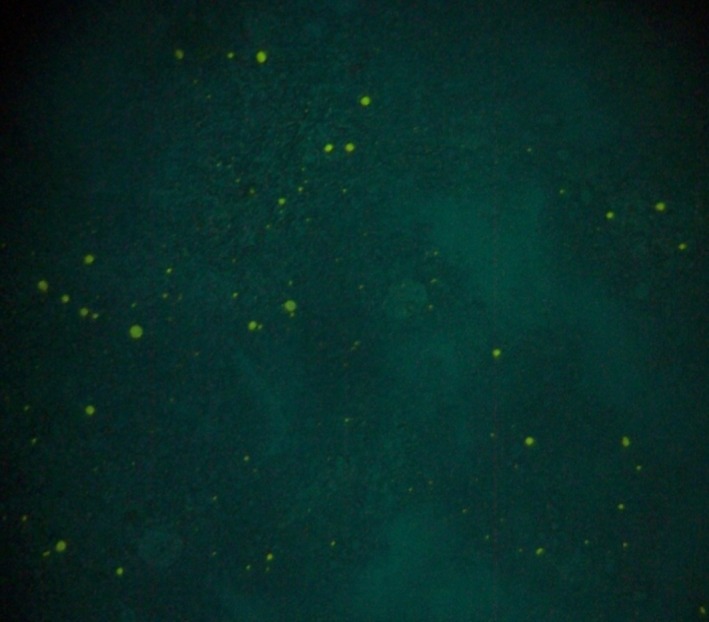
Direct Fluorescent antibody Test (FAT) showing positive result

The test is considered positive when there is an appearance of brilliant apple‐green fluorescence color exhibited against a black background. If no specific apple‐green fluorescence is exhibited, the test is negative (Figure 3).

## 3 DISCUSSION

Rabies has presently been eliminated from domestic dogs in developed countries but remains a nightmare in developing countries. In Nigeria, it is believed that many cases of rabies in livestock go unreported, possibly due to the fact that, the disease is customarily associated with a snarling mad dog, foaming at the mouth. There is little awareness of rabies in livestock especially if it is the dumb form of rabies and the symptoms may easily be mistaken for some other problems such as organophosphate poisoning, choke among others. Unfortunately, the result is often human exposure to the dreadful rabies virus. Most goats are raised in Nigeria mainly by semi‐intensive or free‐range system of management. Most of these goats herd roam about the streets and refuse dumps sites where they are often exposed to bites by stray dogs. They are often presented to the clinic with complaints of inability to eat and therefore serve as a potential source of exposure to the owner, veterinarians, and their assistants who try to help in the examination or treatment of such animal without protective clothing. Hence, the need for general awareness of livestock owners for the need to pay attention to changes in the behavior of their animals as rabies signs could be unpredictable. Similarly, veterinarians should wear latex gloves prior to examination of the mouth of livestock to avoid contact with possibly contaminated saliva.

It has been established that, the closer the bite is to the head, the shorter the incubation period.^3^ In the present case, overt signs of rabies were expected to manifest quite early because the site of bite was closed to the head. The signs in this animal were however delayed. This perhaps may be as a result of the vigorous cleaning of the site of bite with an antiseptic soap and copious amount of water which perhaps may have reduced the viral load. This practice is advocated especially if the bite is far from the head since the virus is slow moving. Though the furious form of rabies is said to be more frequently encountered, the clinical manifestation of the disease in this goat was the dumb form. This is similar to the observation of Tresamol et al^11^ who also reported the dumb form of rabies in a goat.

With regards to the diagnosis of rabies, FAT using the corneal impression smear technique has been found to be rewarding even in an early stage where overt clinical signs are absent. Initial diagnosis of this case was however based on the rapid immunodiffusion diagnostic test (RIDT). This test is simple to perform, rapid, and sensitive. It is suitable for use under field conditions especially in developing countries with limited diagnostic resources. The confirmatory diagnosis was by FAT which is a test that demonstrates the rabies virus nucleoprotein antigen (N) in fresh brain smears. It is an accurate, sensitive, and rapid test recommended by WHO and OIE.^13^

It is advised that, if an exposed animal is to be slaughtered for consumption, it should be done immediately after exposure with the substantial area of bite being excised. Furthermore, barrier precautions such as latex gloves, eye protection, and face shield should be used by persons processing the meat, and all tissues should be cooked thoroughly.^14^

Since rabies is endemic in Nigeria and livestock exposure is invariably due to bites from rabid dogs, routine vaccination of dogs against rabies should be enforced.

## AUTHORSHIP

KBY: received the case when it was presented to the hospital and wrote the first draft of this manuscript. ASW: was the rabies specialist at the hospital who processed the brain sample at the laboratory. IS: assisted in monitoring the goat during the period of hospitalization and gave useful advice on the case. OSO: did the first edition of the manuscript. JBM: did the final edition of the manuscript.

## CONFLICT OF INTEREST

The authors declare that there is no competing interest.
